# From marrow memory to adverse ventricular remodeling: trained immunity after myocardial infarction

**DOI:** 10.3389/fimmu.2026.1962412

**Published:** 2026-09-29

**Authors:** Xuanchun Huang, Lanshuo Hu, Xinxin Shi, Chengzhi Hou, Min Zhu

**Affiliations:** 1 The First Affiliated Hospital of Zhejiang Chinese Medical University (Zhejiang Provincial Hospital of Chinese Medicine), Hangzhou, China; 2 Tongde Hospital of Zhejiang Province, Hangzhou, China; 3 Guang’anmen Hospital, China Academy of Traditional Chinese Medicine, Beijing, China

**Keywords:** cardiac fibrosis, cardiac remodeling, epigenetic reprogramming, immunometabolism, macrophage–fibroblast crosstalk, myocardial infarction, sterile inflammation, trained immunity

## Abstract

Myocardial infarction [MI] triggers a sterile inflammatory response that is essential for necrotic-tissue clearance and scar formation and should normally subside as repair progresses. In a subset of patients, however, persistent myeloid activation accompanies progressive fibrosis, ventricular dilatation, and adverse remodeling long after the acute insult. Trained immunity may help explain this temporal mismatch by allowing myocardial injury to leave a durable functional bias in innate immune cells or their hematopoietic progenitors. Damage-associated molecular patterns, inflammatory cytokines, and sympathetic signals released after MI may reprogram bone-marrow hematopoiesis through metabolic and epigenetic mechanisms. Bone-marrow-derived cells can enter the circulation and injured myocardium directly; the spleen may provide an additional reservoir, hematopoietic niche, and amplification or deployment compartment rather than an obligatory intermediate. Resident and recruited cardiac macrophages then interpret this systemic input within the heart, where macrophage-fibroblast-extracellular matrix interactions influence scar composition, remote-zone fibrosis, ventricular geometry, and progression toward heart failure. This review follows a heart-hematopoietic-heart network and distinguishes direct MI-trained-immunity evidence from MI-specific bridging evidence, adjacent trained-immunity models, and downstream remodeling evidence. Individual components are supported to different degrees, but causal continuity from MI-induced hematopoietic memory to adverse ventricular remodeling has not yet been demonstrated. Defining where durable memory is established, how myeloid output reaches the heart, and when it becomes maladaptive may support phase-specific interventions that preserve early repair while limiting later inflammation and fibrosis.

## 1 Introduction: from transient myocardial injury to persistent ventricular remodeling

### 1.1 Adverse ventricular remodeling after myocardial infarction

#### 1.1.1 Acute inflammation as an essential component of infarct repair

Myocardial infarction [MI] initiates a tightly coordinated sterile inflammatory response that is indispensable for removing necrotic tissue and establishing a mechanically stable scar. Yet the same response can become maladaptive when leukocyte recruitment, cytokine production, or extracellular-matrix turnover persists beyond the period required for repair. This imbalance contributes to infarct expansion, remote-zone fibrosis, ventricular dilatation, and the progression to ischemic heart failure. Experimental and clinical studies have shifted the field away from the view that post-infarction inflammation is uniformly harmful. Inflammatory clearance and reparative transition are both necessary. Their timing, cellular origin, and resolution determine whether healing remains adaptive or progresses toward adverse remodeling (1–3).

#### 1.1.2 Failure of inflammatory resolution and adverse remodeling

Although inflammatory clearance and reparative transition are both necessary, their timing, cellular origin, and resolution determine whether healing remains adaptive or progresses toward adverse remodeling. The central unresolved problem is temporal. Circulating monocytes are short-lived, yet altered myeloid phenotypes, residual inflammatory signals, fibrosis, and ventricular dysfunction can persist for months after the index event. Two human studies approached this problem from different clinical questions. Da Silva et al. prospectively examined whether contemporary lipid-lowering and antiplatelet strategies normalized CCR2, CCR5, and CX3CR1 expression across circulating monocyte subsets during the first six months after acute MI (4). Mol et al. asked whether young patients with premature MI in the absence of standard modifiable risk factors retained monocyte hyperresponsiveness and trained-immunity-associated chromatin changes long after the event; participants were studied a median of approximately 25 months after MI (5).

The first study was designed to characterize the persistence and treatment responsiveness of a proinflammatory monocyte phenotype, rather than to test memory with a rest-rechallenge protocol. The second incorporated ex vivo Toll-like-receptor stimulation and H3K4me3 analysis, but its cross-sectional design could not determine whether the phenotype preceded or followed MI. These complementary goals explain why the studies support a durable post-MI myeloid signal without establishing MI-induced trained immunity. Together, they expose a clinically relevant mismatch between a transient ischemic insult and a persistent bias in innate immune behavior (4, 5).

### 1.2 Trained immunity as a link between acute injury and long-term remodeling

#### 1.2.1 The temporal paradox of post-MI inflammation

The conventional model of post-infarction inflammation explains acute leukocyte recruitment, debris clearance, resolution, and scar formation. It does not fully explain why altered myeloid behavior can outlast the mature cells present during the index event. Continued tissue injury, recurrent ischemia, and neurohumoral activation may sustain inflammation, but they do not exclude a longer-lived change upstream of circulating cells. The question that motivates this review is therefore whether MI changes the response potential of newly generated or tissue-persistent innate cells after the initiating injury has substantially waned.

#### 1.2.2 Trained immunity as an explanatory framework

Trained immunity provides a potential explanation for this temporal gap. It describes persistent functional reprogramming of innate immune cells or their progenitors after an initial stimulus, resulting in an altered response to a later challenge. Unlike adaptive immune memory, it does not depend on antigen-specific lymphocyte receptors. Its durability may instead arise from metabolic and epigenetic reprogramming in long-lived hematopoietic stem and progenitor cells [HSPCs], which can transmit a biased response potential to successive waves of short-lived myeloid progeny (6–10).

This framework is particularly relevant to MI because it connects a local cardiac event with later systemic and tissue responses. Cardiometabolic risk factors may have trained hematopoietic cells before coronary occlusion, in which case MI acts as a powerful heterologous challenge. Alternatively, MI-derived DAMPs, inflammatory cytokines, and sympathetic activation may establish a new program or reinforce a pre-existing one (11). These routes are not mutually exclusive and may vary among patients, but each predicts that later myeloid cells respond from a baseline shaped by exposures beyond the immediate cardiac microenvironment.

The strongest MI-related mechanistic evidence supports a persistent bone-marrow component. Bone marrow from MI-exposed donors transferred an enhanced inflammatory and atherogenic phenotype to recipients, while MI induced KMT5A-dependent H4K20 methylation at the Syk locus and enabled CNBP-SYK amplification after a later metabolic challenge (12). Catecholamine and oxidized-low-density-lipoprotein models further support rest-dependent monocyte hyperresponsiveness and metabolic-chromatin persistence (6, 7). The critical limitation is that the MI marrow-transfer study principally demonstrated accelerated atherosclerosis, not post-infarction ventricular remodeling. Trained immunity is therefore a biologically plausible framework for the remodeling problem, but the decisive connection between MI-induced hematopoietic memory and later cardiac structural change remains unproven.

#### 1.2.3 The heart-hematopoietic-heart network and the conditional role of the spleen

We propose an interorgan network in which the infarcted heart supplies initiating signals and bone-marrow HSPCs store or regenerate a persistent myeloid bias. Bone-marrow-derived monocytes may then reach the circulation and injured myocardium directly. In parallel, a proportion of progenitors and monocytes may enter the spleen, where reservoir release, extramedullary hematopoiesis, and niche-dependent retention can modify the number, composition, and timing of cells available for cardiac recruitment. The spleen is therefore a conditional amplifier and deployment compartment, not an obligatory gate. Resident and recruited cardiac macrophages interpret these systemic inputs within the changing infarct environment, and their interactions with fibroblasts and extracellular matrix [ECM] influence scar composition, tissue mechanics, ventricular geometry, and, potentially, adverse remodeling. Training is considered a shift in the probability, magnitude, or duration of later myeloid responses rather than a fixed M1/M2 phenotype (3).

#### 1.2.4 Scope and narrative structure of this review

This disease-oriented narrative follows myocardial injury, bone-marrow adaptation, direct and spleen-modulated myeloid deployment, cardiac macrophage responses, and macrophage-fibroblast-ECM communication. **Figure 1** depicts the direct marrow-to-blood-to-heart route as the main path and the spleen as a non-obligatory parallel branch. Representative studies supporting individual components are summarized in **Table 1**. The review then integrates the evidence hierarchy, human observations, therapeutic implications, aging, and experiments needed to determine whether durable hematopoietic memory contributes to ventricular remodeling.

**Figure 1:**
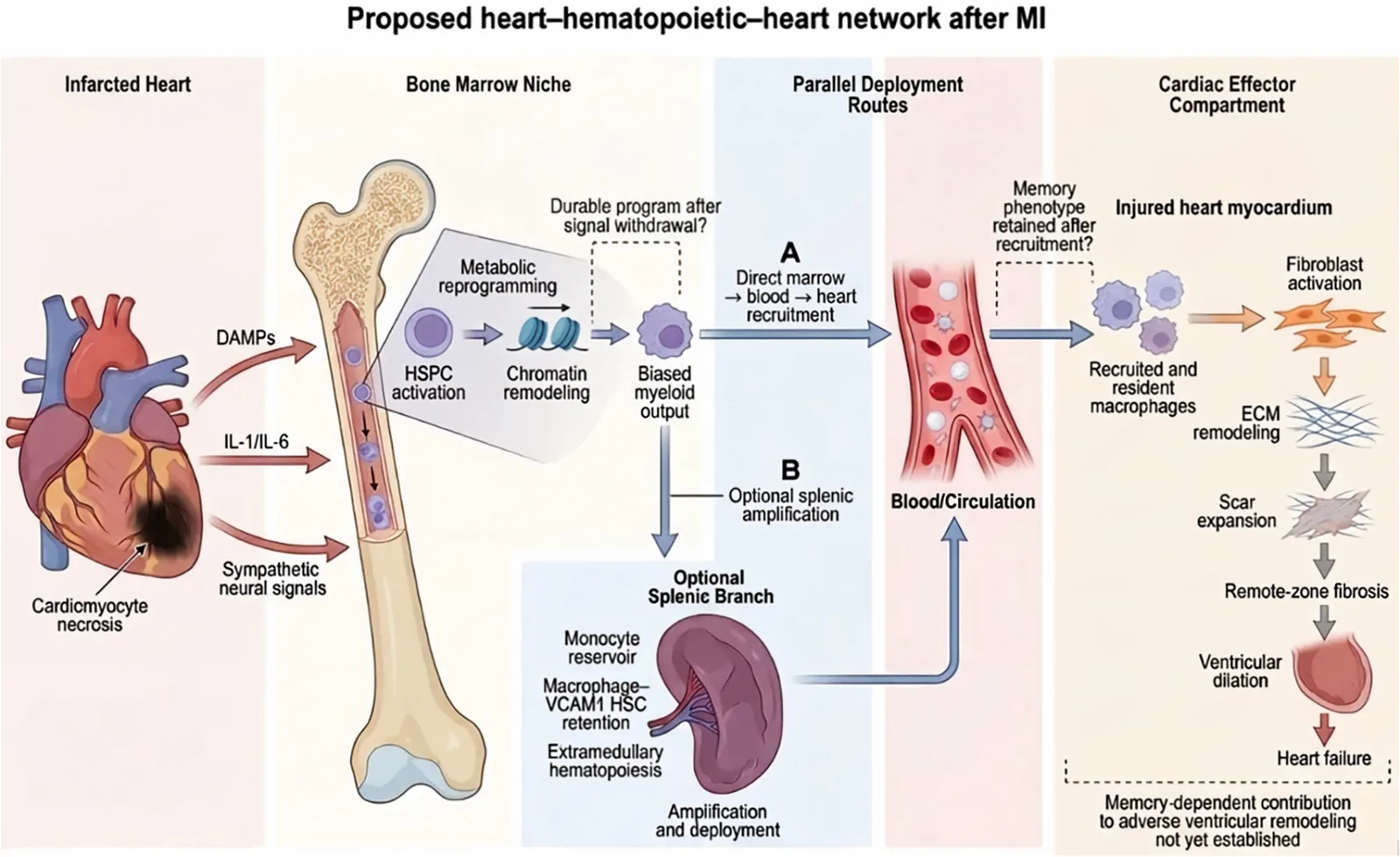
Proposed branched heart–hematopoietic–heart network linking myocardial infarction to adverse ventricular remodeling. MI-derived inflammatory and neurohumoral signals may activate and potentially reprogram bone-marrow hematopoiesis. Bone-marrow-derived myeloid cells can enter the circulation and injured myocardium directly, whereas a parallel, non-obligatory splenic branch may contribute through monocyte reservoir function, macrophage–VCAM1-dependent HSC retention, extramedullary hematopoiesis, and amplification and deployment. Solid arrows denote supported trafficking or component-level mechanisms. Dashed brackets identify unproven memory-specific transitions, including persistence after signal withdrawal, retention of an altered phenotype after cardiac recruitment, and a memory-dependent contribution to adverse ventricular remodeling. DAMPs, damage-associated molecular patterns; ECM, extracellular matrix; HSC, hematopoietic stem cell; HSPC, hematopoietic stem and progenitor cell; MI, myocardial infarction; VCAM1, vascular cell adhesion molecule 1.

**Table 1:** Representative studies supporting components of the post-MI heart-hematopoietic-heart network.

Setting/model	Compartment	Principal observation	Contribution to the proposed pathway	Evidence category	Refs
Treated MI cohort	Circulating monocytes	Persistent chemokine-receptor alterations	Supports prolonged myeloid adaptation after MI	MI-specific human bridging evidence	(4)
Premature MI cohort	PBMC/monocytes	Enhanced ex vivo cytokine response and H3K4me3 signal	Provides a human functional signal of persistent responsiveness	Indirect human trained-immunity evidence	(5)
Experimental MI and marrow transfer	Bone marrow/HSPCs	Transferable inflammatory phenotype; KMT5A-H4K20me-CNBP-SYK axis	Links myocardial injury to durable hematopoietic reprogramming	Direct MI-trained-immunity evidence; remodeling endpoint absent	(12)
Experimental MI	CCR2+ HSPCs	Rapid expansion and myeloid-biased output	Connects cardiac injury with emergency hematopoiesis	MI-specific bridging evidence	(13)
Human post-mortem MI	Marrow-spleen-heart trafficking	Phase-related depletion and cardiac accumulation of monocytes	Supports splenic deployment within interorgan trafficking	MI-specific trafficking evidence	(14)
Fate mapping/single-cell MI studies	Cardiac myeloid cells	Origin- and time-dependent macrophage trajectories	Defines the cardiac execution compartment	Downstream cardiac remodeling evidence	(15–27)
Longitudinal FAPI-PET/CMR	Cardiac fibroblast activity	Fibroblast activation beyond conventional infarct boundaries	Links stromal activation with remodeling phenotypes	Downstream structural evidence	(28)
Experimental MI	Bone marrow-spleen axis	Sympathetic HSPC egress, splenic seeding, reservoir release, and sustained extramedullary monocytopoiesis	Connects ischemic injury with prolonged systemic monocyte supply	MI-specific trafficking and amplification evidence	(29–31, 56, 57)
Mouse and human single-cell MI data	Bone-marrow progenitors and myeloid cells	Type I interferon program detectable in marrow before cardiac entry	Supports remote activation of the hematopoietic compartment after MI	MI-specific hematopoietic bridging evidence	(32)

## 2 The heart-hematopoietic-heart network after myocardial infarction

### 2.1 Signals from the infarcted heart to the hematopoietic system

#### 2.1.1 Damage-associated molecular patterns and sterile inflammation

Necrotic cardiomyocytes and damaged extracellular matrix release a heterogeneous mixture of damage-associated molecular patterns [DAMPs], including HMGB1, extracellular ATP, mitochondrial DNA, S100 proteins, and matrix fragments. These signals activate pattern-recognition receptors [PRRs] on resident cardiac cells, recruited leukocytes, endothelium, and stromal cells. TLR2/4, RAGE, cGAS–STING, ZBP1–IRF3, and inflammasome pathways converge on cytokine, chemokine, adhesion, and cell-death programs that initiate leukocyte recruitment and debris clearance (33–40). This response is biologically necessary; the relevant question is when its magnitude or duration becomes maladaptive.

#### 2.1.2 Cytokine-mediated hematopoietic activation

IL-1 signaling provides a mechanistic link between DAMP sensing, myeloid recruitment, and remodeling. In murine MI, systemic Il1a deletion reduces CCL2, IL-6, VCAM1, Ly6C-high monocyte accumulation, fibrotic gene expression, collagen deposition, and later ventricular dilatation, whereas cardiomyocyte-restricted deletion does not reproduce the long-term benefit (1). Earlier work similarly established a central role for IL-1R1 signaling in infarct healing and remodeling (35). These findings indicate that the IL-1 network integrates multiple cellular sources and cannot be reduced to a direct fibroblast stimulus.

IL-6 is likewise context dependent. It participates in acute-phase signaling, leukocyte communication, and fibroblast regulation, but genetic or pharmacological observations vary with timing, cell source, and model (41, 42). Both IL-1 and IL-6 can communicate local cardiac injury to distant hematopoietic compartments. Nevertheless, cytokine elevation during unresolved injury remains evidence of continued signaling rather than memory unless persistence after signal withdrawal and altered rechallenge are shown.

#### 2.1.3 Sympathetic and neurohumoral signaling

MI activates sympathetic pathways that influence the bone-marrow niche. Experimental evidence links myocardial ischemia to norepinephrine-dependent hematopoietic and monocyte reprogramming, while catecholamine exposure can induce rest-dependent monocyte hyperresponsiveness through beta-adrenergic/cAMP signaling (6, 12, 29). MI also rapidly expands a CCR2-positive HSPC compartment with myeloid bias, although this emergency hematopoietic response does not by itself demonstrate durable trained immunity (13). These observations support three nonexclusive scenarios: cardiovascular risk factors may pre-train myeloid cells before MI; MI-derived DAMPs and sympathetic signals may establish a new memory; or MI may serve as a second challenge that unmasks prior training. **Figure 2** integrates these routes and distinguishes local DAMP amplification from bone-marrow training signals.

**Figure 2:**
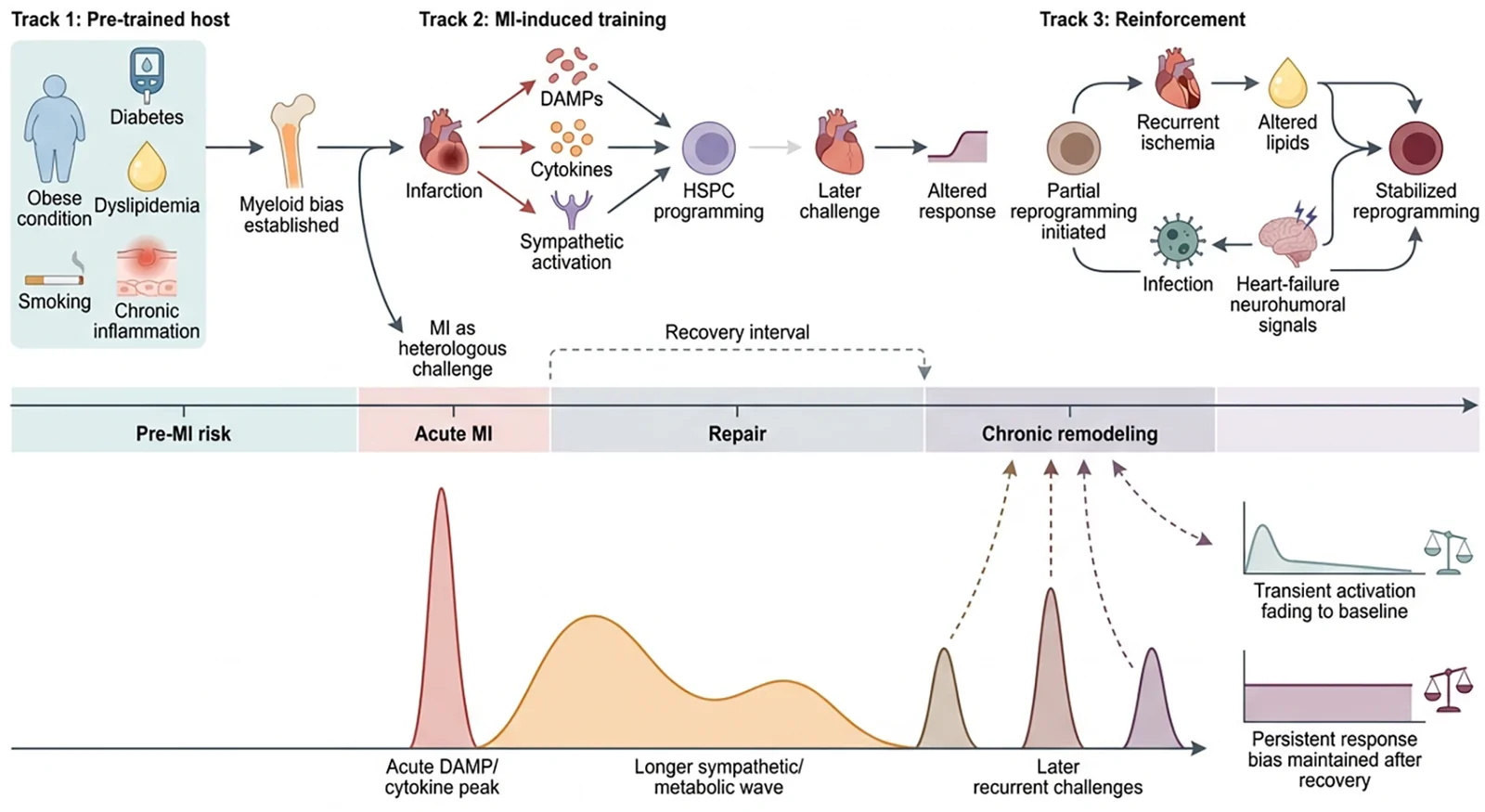
Temporal architectures of persistent myeloid responsiveness after myocardial infarction: myeloid bias may precede MI, arise after MI, or be reinforced by later ischemic, metabolic, infectious, or neurohumoral stimuli. Trained immunity requires persistence after withdrawal of the initiating signal and an altered response after rest or rechallenge; ongoing activation alone is insufficient. HSPC, hematopoietic stem and progenitor cell; MI, myocardial infarction.

#### 2.1.4 Convergence and temporal evolution of systemic signals

The signals generated by MI do not reach hematopoietic tissues as a single synchronized pulse. DAMP and cytokine release rises rapidly with cardiomyocyte necrosis and reperfusion, whereas sympathetic activation, altered lipid handling, recurrent ischemia, and heart-failure-associated neurohumoral stress may persist or recur during repair. Their overlap creates a changing systemic environment in which inflammatory, neural, and metabolic inputs converge on HSPCs and the marrow niche. Consistent with such remote communication, single-cell profiling has detected a type I interferon program in marrow neutrophil and monocyte progenitors before their progeny reach the infarcted heart (32). The biological effect therefore depends not only on signal identity or magnitude, but also on sequence, duration, and the interval between the initial exposure and a later challenge (4–13).

Three temporal routes can account for persistent post-MI myeloid responsiveness. First, cardiometabolic or inflammatory exposures may have trained hematopoiesis before MI, making the infarct a heterologous second challenge. Second, MI-derived DAMPs, cytokines, and sympathetic signals may initiate a new program that persists after acute injury has subsided. Third, the infarct may initiate a partial program that is subsequently reinforced by recurrent ischemia, dyslipidemia, infection, or neurohumoral activation. These routes may coexist, but they are not experimentally interchangeable: pre-existing training requires pre-MI information, MI-induced training requires persistence after withdrawal of the acute stimulus, and reinforcement requires evidence that later inputs stabilize rather than merely accompany the phenotype.

This distinction separates durable reprogramming from prolonged activation. A high cytokine concentration or expanded myeloid compartment during ongoing injury demonstrates systemic signaling, not memory. By contrast, persistence after signal decay, altered function after a defined rest-rechallenge interval, or transfer through a progenitor compartment supports a stored response bias. Initial infarct size, reperfusion, infection, analgesia, and hemodynamic failure must still be controlled because each can increase both systemic inflammation and later remodeling. Thus, the first outward phase of the pathway is well established, whereas the next question is whether convergent post-MI signals are retained within bone-marrow hematopoiesis—the focus of Section 2.2.

### 2.2 Bone-marrow reprogramming and persistent myeloid bias

#### 2.2.1 Hematopoietic stem and progenitor cell activation

Central trained immunity provides a biological explanation for memory that outlives circulating monocytes. Inflammatory and metabolic stimuli can alter HSC/HSPC proliferation, lineage bias, and the functional properties of their progeny (9, 43, 44). MI activates CCR2-positive HSPCs and emergency myelopoiesis, directly linking ischemic injury with progenitor output (13). MI-specific marrow-transfer evidence further shows that an ischemic event can imprint a transferable inflammatory phenotype (12). These observations place HSPCs at the center of the proposed long-term response, although emergency myelopoiesis alone does not establish durable memory.

#### 2.2.2 Immunometabolic reprogramming

Innate immune activation rapidly changes glucose, lipid, amino-acid, and mitochondrial metabolism. In trained-immunity models, enhanced glycolytic capacity, pentose-phosphate-pathway activity, glutaminolysis, cholesterol synthesis, and context-specific mitochondrial remodeling provide energy and biosynthetic intermediates for heightened secondary responses (8, 9, 45–48). mTOR-HIF-1alpha signaling and inflammasome activation can reinforce this state. Metabolites such as acetyl-CoA, fumarate, succinate, S-adenosylmethionine, and lactate also influence chromatin-modifying enzymes or provide substrates for histone modifications, linking transient metabolic adaptation with more durable transcriptional accessibility.

#### 2.2.3 Epigenetic maintenance of altered responsiveness

Metabolic and chromatin changes form a coupled system rather than parallel lists of biomarkers. OxLDL-conditioned monocytes retain H3K4me3 at inflammatory and lipid-handling genes after rest, and inhibition of histone-methyltransferase activity attenuates both enhanced cytokine production and foam-cell formation (7). Catecholamine-trained cells likewise show persistent functional and metabolic changes (6). In HSCs, transient inflammatory exposure can leave C/EBPbeta-dependent accessibility at myeloid enhancers and increase responsiveness to a later challenge (49). A recent human xenograft inflammation-recovery study identified an inflammatory-memory HSC subset [HSC-iM] with durable transcriptional and epigenetic features and transmission of stimulus-shaped programs to downstream myeloid progeny (50). This study closes an important species and lineage-transmission gap by showing that human HSCs can retain inflammatory history after recovery. It does not, however, identify MI as the inducing stimulus or connect HSC-iM to ventricular remodeling. In MI-exposed marrow, KMT5A-dependent H4K20 methylation at Syk and later CNBP-SYK amplification provide a more disease-proximal candidate substrate (12). Other MI studies implicate EZH2-regulated H3K27 methylation, histone lactylation, and NPM1-linked metabolic-epigenetic control in macrophage repair programs (51–53), although most address acute differentiation or repair rather than durable HSPC memory.

The persistence of this coupling depends on how metabolic flux is translated into chromatin-enzyme activity. Increased glycolysis and citrate export can raise cytosolic acetyl-CoA availability and support histone acetylation, whereas changes in one-carbon metabolism influence S-adenosylmethionine-dependent methylation. Accumulation of fumarate or succinate can restrain alpha-ketoglutarate-dependent demethylation, and lactate can participate in histone lactylation or alter redox-sensitive transcriptional programs (8, 9, 45–47, 51–53). These relationships provide a plausible route by which a transient inflammatory or adrenergic exposure could alter the accessibility of enhancers controlling later cytokine, trafficking, and metabolic responses. In long-lived HSPCs, such accessibility would not need to preserve a single mature-cell phenotype; it could instead bias the range of states available to multiple generations of progeny. This distinction is important after MI, where rapid cell turnover and differentiation make persistence of an identical monocyte state unlikely. The mechanistic claim is therefore not that one metabolite or histone mark constitutes memory, but that a coupled metabolic-chromatin program may preserve altered response potential through hematopoietic renewal. Demonstrating this in post-MI remodeling will require concordant changes in metabolite availability, chromatin accessibility, progenitor output, and secondary function, together with perturbation showing that the proposed coupling is necessary rather than merely associated.

These mechanisms cannot be transferred wholesale from cultured monocytes to post-MI HSPCs. Cell division, differentiation, niche cytokines, and substrate availability may reshape the relevant marks. A single post-MI chromatin measurement therefore cannot distinguish durable reprogramming from acute activation or altered cell composition. The KMT5A-H4K20me-CNBP-SYK pathway remains especially important because the marrow phenotype was transferable, but its downstream endpoint was atherosclerosis rather than ventricular remodeling (12).

#### 2.2.4 From progenitor reprogramming to altered myeloid output

The functional consequence of central reprogramming is not the presence of an isolated histone mark but a sustained change in the production or response potential of myeloid progeny. Biased HSPC output may increase the supply of inflammatory monocytes or generate descendants with altered thresholds for cytokine production, efferocytosis, resolution, and repair. Bone marrow is therefore treated as the leading candidate site at which a transient cardiac signal could be converted into persistent myeloid bias. The decisive remodeling experiment remains missing: selective interruption of the putative marrow program must normalize later cardiac immune-stromal responses and ventricular outcomes without merely reducing the initial infarct.

The mechanistic sequence is ordered but not anatomically obligatory: injury-derived signals alter HSPC activity; metabolic changes modify substrates and enzymes that regulate chromatin; persistent accessibility biases progenitor output; and later myeloid cells respond differently after direct recruitment to the heart or after additional modulation in the spleen. This sequence links molecular memory to cell supply while allowing more than one route of deployment.

**Figure 3**; **Table 2** compare candidate metabolic and epigenetic substrates while distinguishing MI-specific observations from adjacent trained-immunity models.

**Figure 3:**
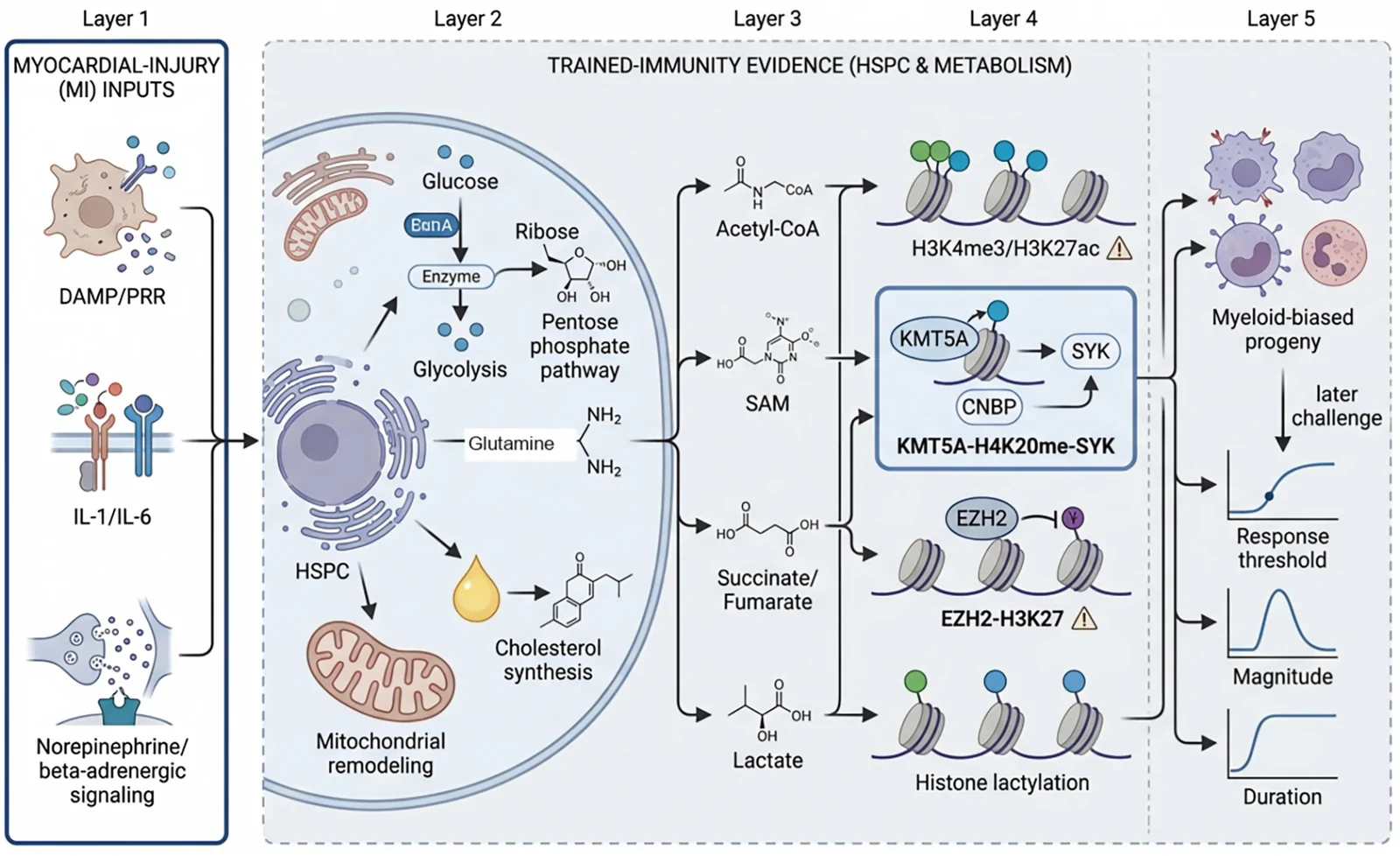
Metabolic–epigenetic coupling as a candidate substrate of post-MI hematopoietic memory: inflammatory and adrenergic signals may alter HSPC metabolism, chromatin regulation, myeloid output, and later response potential. The KMT5A–H4K20me–CNBP–SYK axis provides disease-proximal evidence, whereas the other pathways shown are supported mainly by adjacent trained-immunity or acute repair models. HSPC, hematopoietic stem and progenitor cell; SAM, S-adenosylmethionine.

**Table 2:** Molecular pathways that may establish or reinforce post-MI trained immunity.

Input/pathway	Metabolic or signaling node	Persistent substrate/output	Main compartment	Evidence for durable memory	Refs
MI-DAMP/PRR signaling	TLR2/4, RAGE, cGAS-STING, inflammasome	Cytokine and chemokine programs	Heart and circulating myeloid cells	MI-specific acute signaling; durable memory not shown	(33–40)
IL-1/IL-6 communication	IL-1R1, NLRP3, IL-6 signaling	Myeloid recruitment and hematopoietic instruction	Heart-bone marrow communication	MI-specific communication; hematopoietic instruction is not memory	(1, 35, 41, 42)
Sympathetic activation	Norepinephrine, beta-AR, cAMP	HSPC mobilization and rest-dependent hyperresponsiveness	Bone-marrow niche/monocytes	Mixed MI and adjacent evidence; mobilization must be separated from training	(6, 12, 13)
MI-associated marrow program	KMT5A, H4K20 methylation, CNBP-SYK	Transferable myeloid inflammatory bias	HSPCs and progeny	Direct MI-trained-immunity evidence; transferable phenotype, non-remodeling endpoint	(12)
Oxidized LDL training	ERK/PI3K and metabolic rewiring	H3K4me3 and enhanced rechallenge response	Monocytes/macrophages	Adjacent trained-immunity evidence	(7)
Metabolic-chromatin coupling	Glycolysis, OXPHOS, acetyl-CoA, SAM, succinate	Histone methylation, acetylation and lactylation	Central and peripheral myeloid cells	Adjacent mechanistic evidence; memory requires persistence and secondary function	(43, 45–48, 51–55)
Adjacent central trained-immunity models	IL-1/GM-CSF, C/EBPbeta, myeloid enhancers	Biased myelopoiesis and persistent HSC chromatin accessibility	HSPCs and myeloid progenitors	Adjacent central trained-immunity evidence	(44, 49)
Human inflammatory HSC memory	HSC-iM transcriptional and epigenetic program	Durable program transmitted to myeloid progeny	Human HSCs and descendants	Direct human HSC-memory evidence; not MI-specific	(50)

### 2.3 The spleen as a non-obligatory reservoir and amplification compartment

#### 2.3.1 Splenic monocyte reservoir after myocardial infarction

The spleen occupies a distinct but non-obligatory position in the post-MI network. It is unlikely to be the principal long-term memory reservoir, a role more plausibly assigned to HSC/HSPCs and their niches. Bone-marrow-derived monocytes can enter the circulation and injured myocardium without splenic transit. The spleen provides a parallel route that stores readily deployable monocytes, supports stress-induced extramedullary myelopoiesis, retains progenitors within an inducible vascular niche, and regulates the timing and magnitude of cell release. Its contribution may therefore range from limited to substantial according to disease phase, systemic stress, age, and cardiometabolic context.

Experimental MI studies first identified a splenic reservoir of monocytes that can rapidly exit the organ and accumulate in injured myocardium (30). The reservoir function is also supported by human post-mortem evidence. Across phases of MI, myocardial monocyte accumulation is accompanied by depletion of splenic and marrow monocytes, consistent with coordinated recruitment along a marrow-spleen-heart axis (14). These observations provide anatomical evidence for interorgan trafficking, although they cannot determine whether the mobilized cells were trained, newly produced, or simply responding to persistent chemotactic signals. The relevant interpretation is therefore not that splenic depletion proves immune memory, but that the spleen can supply the cardiac effector population through which an upstream hematopoietic bias might be expressed.

#### 2.3.2 Extramedullary hematopoiesis and myeloid amplification

The spleen is also an active hematopoietic and immunoregulatory site rather than a passive holding compartment. After MI, bone-marrow progenitor egress and splenic seeding can produce a sustained increase in monocyte supply, while extramedullary monocytopoiesis helps maintain rapid monocyte kinetics (29, 31). Splenic red-pulp macrophages contribute directly to this niche: VCAM1-expressing macrophages retain HSCs through VLA4-VCAM1 interactions, and disrupting macrophage maturation or VCAM1 reduces splenic HSC retention, circulating leukocytosis, and inflammation in infarcted myocardium and atherosclerotic plaques (56). In an obesity-hypertension model of HFpEF, fatty-acid metabolism activated red-pulp macrophages, increased VCAM1-dependent HSC retention and proliferation, and established a splenic hematopoietic niche (57). The latter is adjacent rather than MI-specific evidence, but it shows how cardiometabolic stress can convert the spleen into an active amplifier of hematopoietic output.

Lymphocytes provide another layer of regulation. B-cell-derived CCL7 promotes monocyte mobilization, whereas regulatory B-cell programs can restrain migration and improve remodeling (58–60). Conventional CD4 T cells and Treg likewise exert different effects on splenic myelopoiesis and monocyte differentiation (61). These findings clarify why the net splenic effect cannot be labeled uniformly inflammatory or protective; it depends on the niche cell, recruited lineage, signal, and disease phase.

#### 2.3.3 Mobilization of splenic myeloid cells to the infarcted heart

Storage, production, retention, and regulated deployment are related but separable splenic functions. Marrow-derived progenitors or monocytes carrying a persistent response bias may seed or transit through the spleen, where macrophage-VCAM1 retention, local proliferation, and lymphocyte-dependent release modify their abundance and timing before cardiac entry (14, 56–61). Other marrow-derived cells may bypass the spleen and move directly through blood to injured myocardium. Available data establish trafficking and amplification rather than obligatory splenic transit or memory storage.

The relative contribution of these routes likely changes over time. Immediately after infarction, rapid release from an existing splenic monocyte reservoir can supplement direct marrow mobilization. During repair, continued marrow output and stress-induced extramedullary hematopoiesis may replenish or expand the splenic pool, creating later waves of cells with a different developmental history from those recruited during the first inflammatory peak (14, 29–31, 56–61). A marrow-derived response bias could therefore reach the heart directly or be quantitatively and temporally modified in the spleen. Distinguishing these routes requires lineage-resolved tracking and selective perturbation of the splenic niche rather than measurements of splenic size or cell abundance alone.

#### 2.3.4 Functional position of the spleen within a branched interorgan network

The spleen is best positioned as an optional reservoir, niche, and amplification or deployment compartment within a branched network. In patients with acute MI, FDG-PET has demonstrated coordinated metabolic activation of the spleen and bone marrow, providing an *in vivo* signal of cardiosplenic-hematopoietic engagement (62). Nevertheless, splenic size, metabolic activity, monocytosis, or extramedullary hematopoiesis cannot identify cell origin, obligatory transit, or durability of the program. Serial measurements paired with marrow activity, circulating-cell function, and cardiac imaging are more informative than a single splenic readout. Marrow remains the leading candidate site of durable storage; blood provides a direct route to the heart; the spleen can reshape cell supply and timing; and the heart determines how systemic input is expressed within injured tissue.

Neural, inflammatory, and metabolic signals can coordinate marrow and splenic compartments. Sympathetic fibers and stromal, endothelial, osteolineage, and macrophage niche cells regulate progenitor retention and output, while cytokines and autonomic signals influence splenic hematopoiesis and trafficking (9, 12, 13, 43, 56, 57, 64, 65). Cell-intrinsic reprogramming and continued niche instruction may coexist. The spleen is therefore neither an independent memory organ nor a required conduit; it is a context-dependent compartment capable of retaining, expanding, filtering, and temporally reshaping hematopoietic output.

### 2.4 Cardiac macrophages as local interpreters of systemic myeloid bias

#### 2.4.1 Resident and recruited macrophages after myocardial infarction

The infarcted heart contains resident macrophages and successive waves of recruited monocytes that differ in origin, replenishment, spatial distribution, and function (3, 15–17, 54, 55). Early neutrophil and Ly6C-high monocyte influx supports debris clearance and inflammatory amplification; later monocyte/macrophage states contribute to efferocytosis, angiogenesis, matrix regulation, and scar maturation (18–26). Fate-mapping and single-cell studies further show that recruited monocytes generate spatially restricted macrophage and dendritic-like trajectories that can persist in the myocardium (27). These states form a continuum and should not be collapsed into a static M1/M2 dichotomy.

#### 2.4.2 Recruitment, proliferation, and persistence in the cardiac compartment

CCR2-dependent recruitment is especially important for inflammatory monocyte entry, while resident macrophage loss and local proliferation alter the composition of the cardiac niche. In chronic ischemic heart failure, both recruitment and proliferation can maintain an expanded macrophage compartment (66). Multi-modal single-cell profiling and fate mapping indicate that some monocyte states are specified before or during recruitment, whereas Trem2-high and other reparative programs are acquired within the ischemic tissue (22, 23, 27). A trained marrow could increase the supply or responsiveness of recruited cells, but local hypoxia, dying cells, cytokines, and matrix signals continue to shape their final phenotype. Thus, central training changes probability, not destiny.

#### 2.4.3 Myeloid bias and the trajectory of macrophage responses

Post-MI macrophage heterogeneity should be represented along at least four axes: developmental origin, time after injury, anatomical location, and functional state. Resident and recruited populations may converge transcriptionally while retaining different proliferative histories or epigenetic constraints. Conversely, cells of the same origin can diverge under hypoxia, efferocytosis, matrix stiffness, lymphocyte-derived cytokines, and neurohumoral signals (3, 54, 55). These dynamics make the M1/M2 dichotomy particularly unsuitable for testing trained immunity, because it collapses transient state, lineage, and function into a single label.

Training is better conceptualized as a change in the probability of entering particular states after stimulation. A centrally trained progenitor may generate monocytes that are more likely to adopt inflammatory or profibrotic programs, without determining their final fate. Local IL-10, regulatory lymphocytes, apoptotic-cell uptake, and reparative matrix signals may still redirect those cells (2, 67–74). TREM2-dependent metabolic reprogramming and macrophage-derived VEGFC provide direct examples of how efferocytosis can couple clearance to repair, while CD40-TRAF2/3/5 and neutrophil IRGM1 define additional candidate control points (69–72). This probabilistic model explains why a durable upstream bias can coexist with substantial tissue-level plasticity and why single-time-point clustering cannot by itself establish immune memory.

This probability shift can take several nonexclusive forms. Trained progeny may require less stimulation to enter an inflammatory program, produce a larger response at the same stimulus intensity, remain in that state for longer, or recover differently before a subsequent challenge. None of these possibilities requires all recruited monocytes to converge on one stable phenotype. A biased cell entering the hypoxic infarct core may prioritize debris sensing and inflammatory amplification, whereas a related cell entering a vascularized border zone may acquire angiogenic or reparative features; cells reaching remote myocardium encounter still different mechanical and neurohumoral cues (22, 23, 27, 54, 55). The predicted effect of central training is therefore a change in the distribution and duration of cardiac macrophage states across regions and time, not the appearance of a unique trained-macrophage cluster. This view also explains why local IL-10, regulatory lymphocytes, efficient efferocytosis, or reparative matrix signals could partly override an upstream bias (2, 67–74).

Efferocytosis illustrates how lineage history and tissue instruction can converge on a function directly relevant to remodeling. Uptake of apoptotic neutrophils and cardiomyocyte debris is not merely a disposal process: it changes macrophage metabolism, lipid handling, cytokine production, and the release of pro-resolving or angiogenic mediators. TREM2-dependent metabolic adaptation, VEGFC production, CD40-TRAF signaling, and mitochondrial competence each influence this transition (69–73). A trained inflammatory bias could impair the switch from debris sensing to resolution, but defective efferocytosis could also arise entirely from the hypoxic, lipid-rich infarct environment. A memory contribution requires more than a clearance defect within the infarct environment. The altered response should persist after cells are removed from that environment, follow a defined lineage, and normalize when the upstream training mechanism is interrupted. This distinction is important because two macrophages with similar transcriptional states may have arrived there through different histories and may respond differently to a later challenge.

#### 2.4.4 Adaptive regulation, inflammatory resolution, and repair

Treg, conventional CD4 and CD8 T cells, B cells, and dendritic cells alter myeloid recruitment and differentiation. Foxp3-positive CD4 T cells improve healing partly by modifying monocyte/macrophage differentiation (67, 75–77). Delivered Treg adopt an injury-specific cardiac phenotype, reduce CCR2-positive inflammatory myeloid cells, and lose therapeutic benefit when macrophages are depleted or Treg-derived IL-10 is absent (78). CD8 T cells and dysfunctional proinflammatory Treg can instead worsen chronic remodeling (79, 80). **Figure 4** therefore organizes post-MI macrophages along time, origin, state, and regulatory context rather than binary polarization. In the heart–hematopoietic–heart network, these cells are the candidate executors of an upstream myeloid bias. However, macrophage heterogeneity after MI is well established whereas the fraction specifically attributable to prior training remains unresolved; tissue state alone should not be treated as proof of memory.

**Figure 4:**
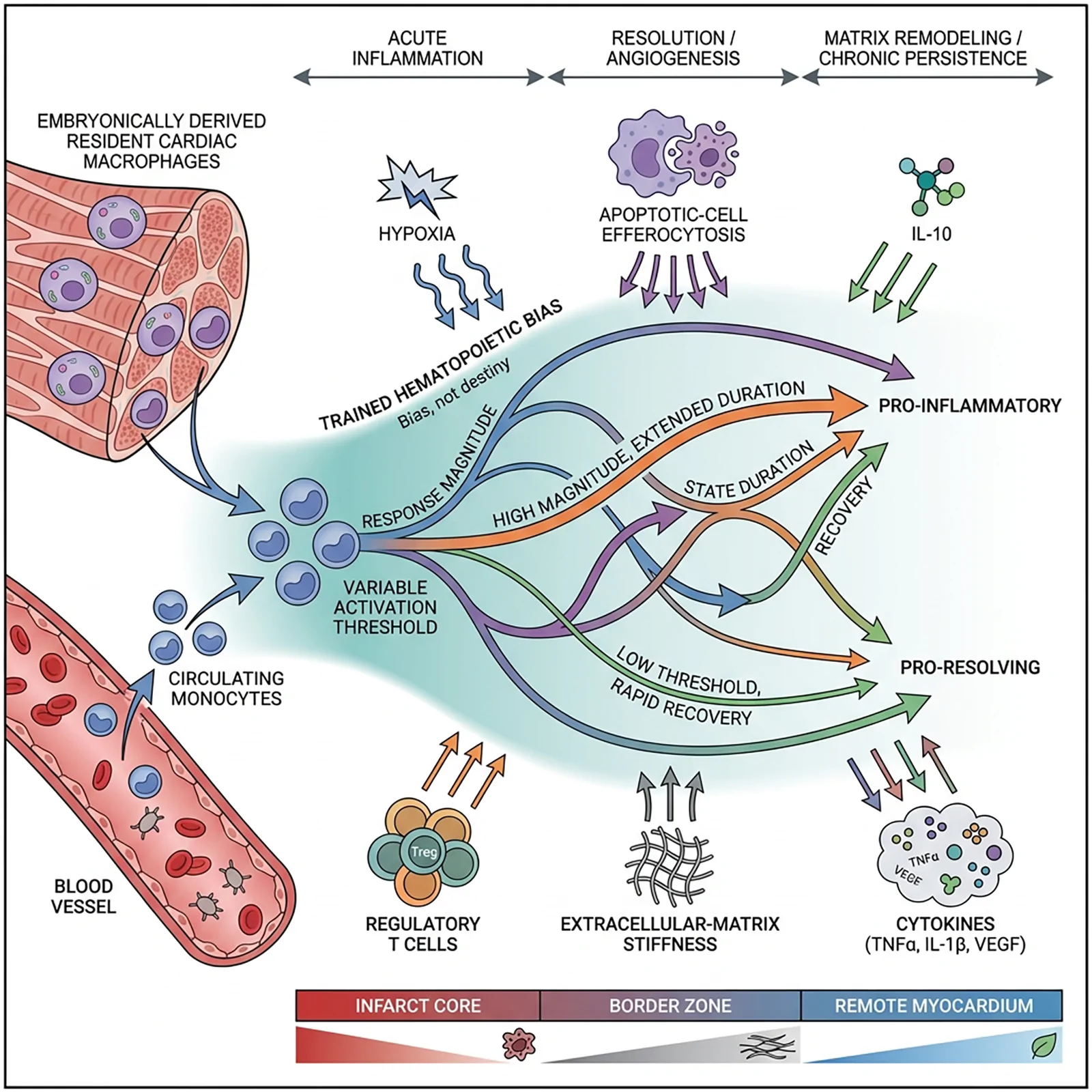
Cardiac macrophages as local interpreters of systemic myeloid bias: resident and recruited macrophages follow origin-, time-, and region-dependent trajectories after MI. A putative hematopoietic imprint may shift response probability, magnitude, or duration, while hypoxia, efferocytosis, regulatory lymphocytes, cytokines, and matrix properties continue to shape cell state. The scheme does not imply a fixed trained-macrophage subtype. MI, myocardial infarction; Treg, regulatory T cell.

The central prediction is probabilistic rather than deterministic. A marrow imprint need not create a fixed macrophage phenotype; it may increase the likelihood, magnitude, or duration of particular responses when recruited cells encounter hypoxia, apoptotic cells, cytokines, and matrix cues. Resident macrophages may carry locally maintained states, whereas recruited monocyte-derived macrophages provide the most direct route by which central hematopoietic bias could reach the heart. Lineage-resolved studies are needed to separate these routes.

### 2.5 The macrophage-fibroblast-extracellular matrix axis in ventricular remodeling

#### 2.5.1 Macrophage-fibroblast signaling

Fibroblast activation is necessary for scar formation but harmful when excessive or sustained in remote myocardium. Macrophages regulate fibroblast proliferation, migration, myofibroblast differentiation, collagen production, and matrix turnover through cytokines, growth factors, extracellular vesicles, matricellular proteins, and efferocytosis-dependent programs (3, 74, 81–88). Recent lineage-tracing data argue against wholesale macrophage-to-fibroblast conversion while showing that macrophages remain major sources of TGF-beta1, PDGFB, osteopontin, thrombospondin-1, fibronectin, and other fibrogenic mediators (83). This directly challenges earlier reports of macrophage–myofibroblast transition and requires that conversion be treated as a disputed mechanism rather than consensus (86). The effect of a given mediator depends on its source and timing.

IL-1 signaling promotes inflammatory recruitment and adverse remodeling, but IL-1 does not simply reproduce TGF-beta/SMAD activation in fibroblasts, indicating an important indirect component (1, 35). IL-10 reshapes macrophage transcription and secretory output, promotes fibroblast migration, alters hyaluronan metabolism, and reduces collagen-I-dominant matrix accumulation (2, 68). IL-27 provides a contrasting candidate pathway: macrophage-associated IL-27 correlates with fibrosis and can stimulate fibroblast proliferation, migration, alpha-SMA, collagen, and JAK2/STAT3 signaling *in vitro*, although *in vivo* neutralization evidence is missing (89).

#### 2.5.2 Fibroblast activation and extracellular matrix turnover

Scar formation prevents cardiac rupture, whereas sustained, excessive matrix deposition and remote myocardial fibrosis elevate myocardial stiffness and drive ventricular dilatation. Though alternatively activated macrophage subsets facilitate connective tissue remodeling, angiogenesis and infarct repair (90–93), the generic label “reparative macrophage” is misleading, as many of these macrophage populations carry intrinsic profibrotic properties. Distinct signaling axes, including ECM1, ITIH5-dependent macrophage-fibroblast crosstalk, VSIG4-expressing macrophages, and the IL-10–STAT3–galectin-3 cascade, mediate divergent immune signals that tune fibroblast activity and matrix synthesis (74, 84, 87, 88, 94). Activated fibroblasts also secrete large quantities of high-molecular-weight hyaluronan, a non-collagen matrix component that accumulates long after acute myocardial infarction (MI) (94). Accordingly, therapeutic strategies should not aim to globally suppress fibroblast activation; the core goal is to preserve early scar stabilization while blocking prolonged, ectopic matrix deposition in non-infarcted remote myocardium.

Macrophage-fibroblast signaling operates bidirectionally, rather than in a one-way unidirectional manner. Fibroblasts are not merely passive responders to macrophage-derived cues. Upon activation, they secrete chemokines, inflammatory cytokines, and matricellular fragments that govern monocyte recruitment, macrophage retention, and local macrophage polarization. Progressive collagen deposition and rising tissue stiffness trigger robust mechanotransduction cascades and remodel extracellular ligand pools, which reciprocally reshape the functional profiles of both fibroblasts and cardiac macrophages. This self-sustaining feedback loop can perpetuate inflammation and fibrotic remodeling long after the initial necrotic myocardial stimulus has resolved. This locally persistent fibrotic phenotype can easily be confused with the systemic trained immunity phenotype originating from bone marrow, yet it arises from a distinct form of tissue-level ecological memory stored within intercellular crosstalk and ECM architecture. To experimentally disentangle hematopoietic cellular memory from matrix-driven fibrotic reinforcement, investigators must perform reciprocal myeloid cell transplantation experiments using standardized cardiac microenvironments with defined ECM characteristics.

Extracellular matrix (ECM) exerts regulatory effects via both biochemical composition and three-dimensional physical architecture. Total collagen content alone is insufficient to predict whether a scar confers mechanical protection or drives maladaptive remodeling. Instead, fiber alignment, collagen crosslink density, retention of provisional matrix, proteolytic fragment generation, and spatial distribution of hyaluronan and matricellular proteins collectively dictate tissue porosity, stiffness, and ligand availability. These biophysical and biochemical signatures modulate integrin ligation, focal adhesion signaling, cytoskeletal tension, and mechanosensitive transcription programs in fibroblasts; meanwhile, soluble matrix fragments and sequestered growth factors continuously recruit and activate circulating myeloid cells (74, 81–88, 94, 95). Extensively stiffened, disorganized ECM can independently lock fibroblasts in a hyperactivated state and retain inflammatory macrophages, even in the absence of continuous myeloid input from the bone marrow. In contrast, sustained delivery of epigenetically biased myeloid progeny (shaped by post-MI trained immunity) amplifies this self-perpetuating matrix loop by altering protease secretion, collagen turnover, and matricellular protein expression. These two pathological pathways operate simultaneously yet can be experimentally dissociated: hematopoietic memory originates upstream within long-lived HSPCs and their myeloid progeny, while matrix-mediated fibrosis is an autonomous, tissue-resident program confined to cardiac tissue. The coexistence of these two mechanisms explains why identical systemic myeloid bias can produce divergent fibrotic outcomes across infarct core, border zone and remote myocardium, and why comparable total collagen loads are often accompanied by drastically different ventricular mechanical function.

The spatial organization of scar tissue carries equal or greater functional importance than total collagen abundance. Transient early matrix deposition restricts infarct expansion and ventricular rupture, yet excessive collagen crosslinking, persistent provisional matrix accumulation, and widespread fibrosis in intact remote myocardium impair global ventricular compliance and propagate abnormal mechanical stress across the entire heart. Alongside fibrillar collagen, non-collagen molecules including hyaluronan, fibronectin, osteopontin and thrombospondin modulate leukocyte migration, growth factor bioavailability, and inflammatory resolution (74, 83–88, 94, 95). A myeloid cell imprint established via post-MI trained immunity may remodel the temporal kinetics and regional distribution of these matrix molecules, without necessarily causing a simple uniform increase in total collagen volume. This refined matrix-centric framework bridges innate immune cell function and whole-organ ventricular remodeling, and it warns against relying solely on collagen quantification as the primary therapeutic endpoint for trained immunity-targeted interventions.

Collectively, reciprocal macrophage-fibroblast-ECM crosstalk positions the extracellular matrix as an active signaling hub, rather than a static, inert end-product of post-infarction inflammatory responses. Macrophage-derived mediators control fibroblast proliferation and matrix biogenesis, while ECM composition, tissue stiffness and matrix-trapped signaling molecules feed back to modify macrophage and fibroblast functional trajectories. **Table 3** summarizes the major immune-stromal communication axes and their corresponding remodeling phases and pathological outcomes. Nevertheless, a critical unresolved question remains within this signaling network: it has not been validated whether MI-triggered hematopoietic trained immunity stably imprints a profibrotic transcriptional signature onto cardiac macrophages to drive persistent maladaptive ECM remodeling.

**Table 3:** Cellular interactions linking myeloid adaptation to scar formation and ventricular remodeling.

Regulatory cell/source	Target	Communication axis	Predominant phase/outcome	Refs
Inflammatory myeloid cells	Endothelium and fibroblasts	IL-1, CCL2, IL-6, adhesion pathways	Early recruitment and later adverse remodeling	(1, 33–42)
Macrophages	Fibroblasts	IL-10-regulated secretome and hyaluronan metabolism	Resolution, fibroblast migration and matrix maturation	(2, 68)
Macrophages	Fibroblasts/ECM	TGF-beta1, PDGFB, osteopontin, thrombospondin-1	Myofibroblast activation and matrix deposition	(81–89)
Treg	Monocytes/macrophages	IL-10 and CCR2-related regulation	Reparative transition and reduced inflammatory recruitment	(67, 75–78)
B-cell subsets	Splenic and circulating monocytes	CCL7 and regulatory signals	Monocyte mobilization or restraint	(58–60)
Fibroblasts/ECM	Monocytes/macrophages	Chemokines, matricellular proteins and mechanotransduction	Cell retention, state reinforcement and remote fibrosis	(83–95)

#### 2.5.3 Spatial matrix remodeling and ventricular mechanics

Adverse remodeling integrates infarct expansion, wall thinning, chamber dilatation, remote-zone fibrosis, hypertrophy, and declining systolic function. Persistent inflammation can contribute through continued leukocyte recruitment, protease activity, fibroblast activation, microvascular dysfunction, and neurohumoral reinforcement (96–98). Once heart failure develops, sympathetic and inflammatory signals may feed back to hematopoietic compartments, creating a self-reinforcing loop consistent with, but not necessarily diagnostic of, trained immunity.

The spatial distribution of remodeling provides an additional link between immune activity and ventricular mechanics. Changes confined to the infarct influence wall thickness, tensile strength, and the risk of expansion or rupture, whereas inflammation and fibrosis in the border and remote zones affect global compliance, contractile synchrony, and chamber dilatation. Recruited myeloid cells encounter different oxygen tension, matrix stiffness, vascular supply, and cardiomyocyte stress across these regions, making a uniform cardiac macrophage response unlikely. A persistent systemic bias might therefore be expressed differently in infarct, border, and remote myocardium. Spatial transcriptomics, multiplex imaging, and region-specific matrix analysis are needed to determine whether putatively trained myeloid states colocalize with fibroblast activation and later structural deterioration rather than merely accumulating in damaged tissue (96–98).

#### 2.5.4 From scar formation to adverse ventricular remodeling

Ventricular remodeling is also shaped by hemodynamic and neurohumoral forces that can obscure immune causality. Infarct size, reperfusion success, microvascular obstruction, loading conditions, renal dysfunction, and renin–angiotensin–aldosterone or sympathetic activation all influence chamber geometry and fibrosis. These factors may act both as confounders and as components of the proposed feedback loop: worse remodeling increases neurohumoral activation, which can further stimulate hematopoiesis and immune-cell trafficking. Demonstrating that trained immunity contributes independently therefore requires comparable initial injury and adjustment for subsequent loading and treatment, together with interventions that modify the memory pathway without simply improving the acute infarct.

This final layer converts immune-state probabilities into structural outcomes. The relevant endpoint is not collagen abundance alone, but the timing, composition, cross-linking, spatial distribution, and mechanics of scar and remote-zone matrix. **Figure 5** integrates the candidate route from biased myeloid responses to fibroblast state, ECM organization, ventricular geometry, and function. The downstream immune-stromal literature is strong, but most studies do not measure an upstream marrow-memory state; this is the principal point at which the two experimental traditions remain disconnected.

**Figure 5:**
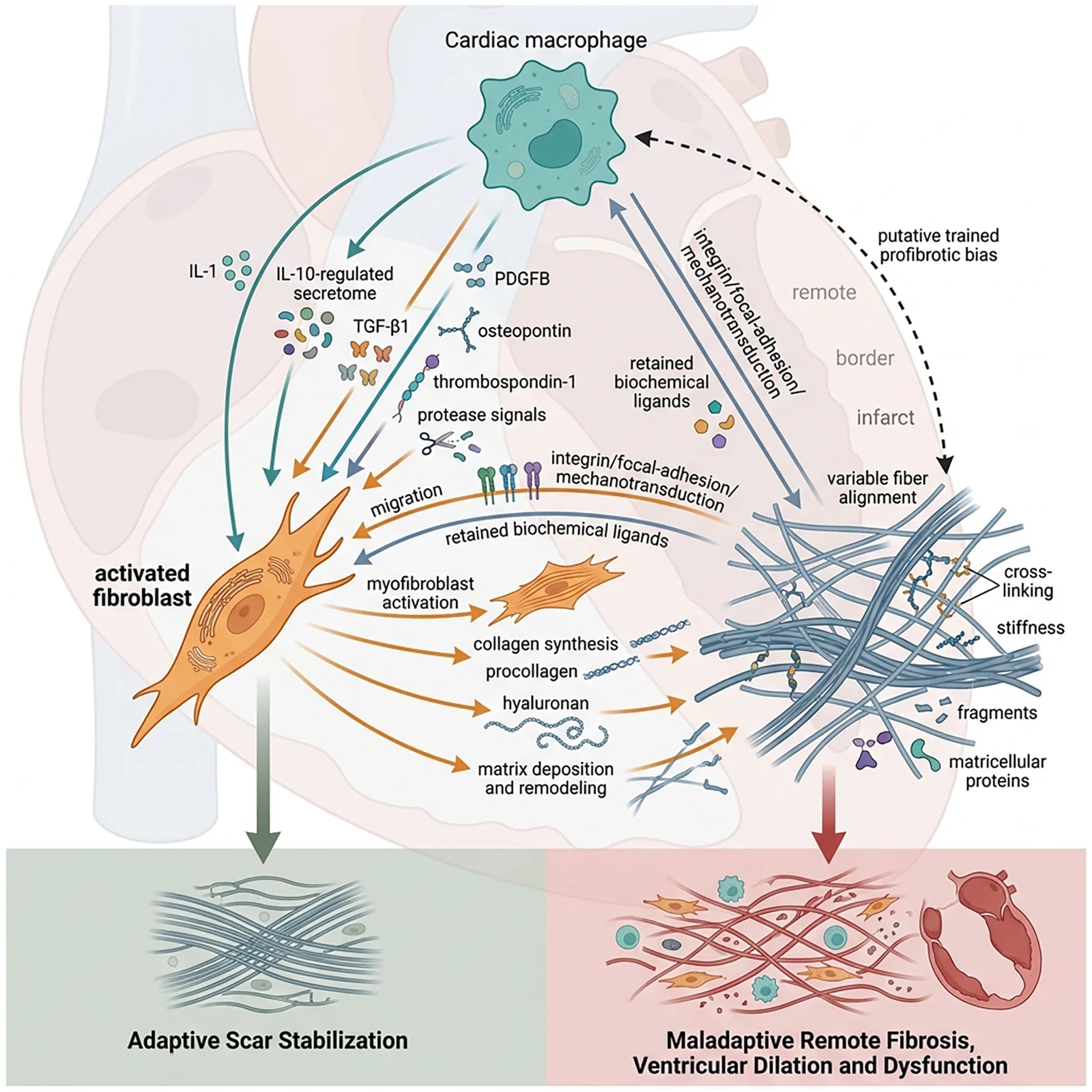
Macrophage–fibroblast–extracellular matrix interactions linking myeloid bias to ventricular remodeling: macrophage-derived signals regulate fibroblast activation and matrix production, while matrix composition and mechanics feed back on both cell types. This network may support adaptive scar stabilization or, when prolonged and spatially inappropriate, promote fibrosis, ventricular dilatation, and dysfunction. The dashed route denotes a putative trained profibrotic bias. ECM, extracellular matrix.

## 3 Evidence supporting the interorgan pathway

### 3.1 Evidence supporting individual interorgan handoffs

To avoid treating unlike observations as equivalent, the following synthesis uses four contextual evidence categories. Direct MI-trained-immunity evidence requires persistence after the acute stimulus, altered secondary function or transferability, and an MI exposure. MI-specific bridging evidence establishes communication, emergency hematopoiesis, trafficking, or remodeling after infarction without demonstrating durable memory. Adjacent trained-immunity evidence establishes memory mechanisms in another stimulus or cardiovascular setting. Downstream remodeling evidence defines cardiac macrophage, fibroblast, ECM, or ventricular effects without measuring an upstream memory state. These labels organize the narrative and **Tables 1**, **2**, **4**; they are not a numerical score and do not interrupt each mechanistic paragraph.

**Table 4:** Therapeutic opportunities across the trained-immunity life cycle.

Disease phase	Memory stage	Target or strategy	Primary compartment	Mechanistic class	Main risk or required endpoint	Evidence status	Refs
Before MI or high-risk state	Pre-training or susceptibility	Cardiometabolic risk and sympathetic input	Systemic and marrow	Risk-factor modification; not memory-specific	Do not claim memory erasure without functional testing	Clinical risk management plus adjacent mechanistic evidence	(6, 9–13)
Acute injury, hours to days	Memory establishment	DAMP/PRR, IL-1/NLRP3, sympathetic signaling	Heart-to-marrow communication	Broad anti-inflammatory or putative establishment-directed	Preserve debris clearance, scar stability, host defense, and survival	Experimental MI; memory-specific engagement generally unproven	(1, 13, 35, 39–42, 103, 104)
Subacute repair, days to weeks	Maintenance and deployment	Metabolic or epigenetic dependencies; myeloid-biased hematopoiesis; splenic niche	Marrow and optional spleen	Trained-immunity-directed candidate	Show reversal of rest-rechallenge phenotype or progenitor bias; monitor cytopenia and infection	Direct MI evidence for selected pathways plus adjacent central-memory and splenic-niche evidence	(6, 7, 12, 43–57)
Reparative to chronic phase	Cardiac effector expression	Efferocytosis, regulatory lymphocytes, IL-10 programs, local RNA or nanoparticle delivery, fibroblast and ECM pathways	Heart	Downstream effector or ECM strategy; not memory-specific	Match start time to scar maturity; assess matrix organization, rupture, remote fibrosis, and ventricular function	Experimental MI and downstream remodeling evidence	(60, 68–78, 105–107)
Secondary prevention after MI	Broad inflammatory reinforcement	Colchicine-related inflammasome suppression	Systemic	Broad anti-inflammatory treatment	Trial heterogeneity; distinguish recurrent ischemic events from structural remodeling	Clinically tested, without demonstrated trained-immunity-specific target engagement	(108–115)

This evidence structure also clarifies what is genuinely new in the trained-immunity interpretation. Conventional post-MI biology already explains acute leukocyte recruitment, splenic mobilization, macrophage heterogeneity, fibroblast activation, and scar remodeling. The additional proposition is one of temporal continuity: myocardial injury may durably alter the production or response potential of subsequent myeloid waves. Cells arriving after the acute inflammatory peak may therefore begin from a different functional baseline and respond differently to signals within the healing myocardium. The trained-immunity framework does not replace established inflammatory and reparative mechanisms; rather, it asks whether persistent hematopoietic adaptation modifies their magnitude, timing, spatial distribution, or recurrence.

No single study currently demonstrates the complete sequence from myocardial injury through durable hematopoietic adaptation and splenic deployment to altered cardiac macrophage behavior and adverse ventricular remodeling. Instead, the model rests on converging evidence from experimental and human studies that illuminate different handoffs along this axis. Its value lies not in assuming that every link has already been proven, but in organizing otherwise separate observations into a temporally coherent and experimentally testable disease process.

The first handoff—from the injured heart to hematopoietic adaptation—is supported by evidence of post-MI HSPC activation, sympathetic regulation of the bone-marrow niche, increased production and mobilization of inflammatory myeloid cells, and transferability of selected marrow phenotypes (12, 13, 29, 32). Marrow-transfer experiments are particularly important because marrow obtained from MI-exposed donors conveyed an enhanced inflammatory phenotype to recipients, accompanied by KMT5A-dependent H4K20 methylation and CNBP–SYK signaling (12). However, the principal downstream phenotype in that study was accelerated atherosclerosis rather than ventricular remodeling. Likewise, post-MI activation of remote hematopoietic compartments and type I interferon-associated marrow responses establishes communication between tissue injury and hematopoiesis, but does not by itself demonstrate durable immune memory (29, 32). Catecholamine- and oxidized-LDL-based training models provide complementary mechanistic support for rest-dependent hyperresponsiveness and metabolic–chromatin persistence (6, 7). These studies therefore strengthen the biological plausibility of the first handoff while remaining adjacent, rather than MI-remodeling-specific, evidence.

The second handoff concerns how altered hematopoiesis supplies cardiac myeloid cells. Bone-marrow-derived monocytes may reach blood and injured myocardium directly, while the spleen provides an additional route through reservoir release, progenitor retention, extramedullary production, and regulated deployment (14, 29–31, 56–62). Splenic macrophage-VCAM1 signaling provides a defined retention mechanism, and lymphocyte-chemokine pathways regulate release (56–61). These findings establish trafficking and amplification but do not show that splenic transit is obligatory or that the spleen stores long-term memory. The revised network therefore predicts variable contributions from direct marrow-to-heart recruitment and spleen-modulated deployment.

The third handoff—from altered myeloid supply to structural remodeling—is supported by fate-mapping, single-cell, and immune–stromal studies. Recruited and resident macrophages follow origin-, location-, and time-dependent trajectories rather than a fixed inflammatory–reparative dichotomy (15–27). Once within the heart, these cells regulate efferocytosis, angiogenesis, fibroblast activation, matrix degradation, collagen deposition, cross-linking, and scar maturation through IL-1-, IL-10-, and TGF-β-related programs, growth factors, matricellular proteins, and interactions with regulatory lymphocytes (1, 2, 66–89). This literature firmly establishes macrophage–fibroblast control of infarct healing and ventricular remodeling. However, most of these studies do not simultaneously measure an upstream marrow or splenic memory state. Consequently, the final connection between durable hematopoietic adaptation and altered cardiac remodeling remains biologically plausible but incompletely demonstrated.

Taken together, the network is supported with unequal levels of directness. Post-MI hematopoietic activation, direct and splenic myeloid deployment, and macrophage-fibroblast regulation of remodeling are individually well established, whereas durable memory and causal continuity connecting them remain less certain. The central unresolved question is whether MI creates or reinforces a hematopoietic program that continues to shape cardiac repair after the initiating inflammatory episode has subsided, and whether the spleen amplifies this process in some settings rather than serving as a universal intermediate.

### 3.2 The missing causal continuity from hematopoietic memory to ventricular remodeling

Evidence is strongest within individual compartments and weakest at the causal transitions between them. A decisive test requires three linked gates: MI must create a progenitor program that persists after the acute signal wanes; descendants of that program must retain altered function as they enter blood and, when relevant, the spleen; and selective interruption of the program must normalize cardiac macrophage-fibroblast communication and improve later remodeling without merely reducing the initial infarct. Emergency hematopoiesis, splenic monocytosis, and cardiac fibrosis therefore support different portions of the story and should not be treated as interchangeable evidence of trained immunity.

The three gates define where added evidence would be most informative. Gate 1 requires an MI-derived progenitor program that survives resolution of acute injury (6, 7, 12, 13, 50). Gate 2 requires lineage-resolved tracking showing that descendants retain altered function during direct marrow-to-blood-to-heart recruitment or after splenic amplification, with selective splenic perturbation used to test rather than assume its necessity (14, 29–31, 56–62). Gate 3 requires evidence that these descendants change cardiac macrophage trajectories and thereby modify fibroblast behavior, ECM organization, and ventricular mechanics (1, 2, 15–27, 66–89). Evidence for all gates should arise within the same experimental sequence and converge on organ-level outcomes.

The clinical timeline can accommodate pre-existing training, MI-induced training, and later reinforcement. Cardiometabolic or inflammatory exposures may bias hematopoiesis before MI; the infarct may establish a new program; and recurrent ischemic, infectious, metabolic, or neurohumoral signals may reinforce that program during recovery (4–7, 9, 11–13). Human imaging observations of marrow activity, remote myocardial inflammation, and fibroblast activation fit portions of this sequence but remain compartment-specific readouts rather than proof of the entire mechanism (63, 99, 100). The pathway is therefore a coherent synthesis of convergent evidence, not yet an established uninterrupted causal cascade. Further discussion concerning experimental testing and translational implications of these validation gates will follow in subsequent sections.

## 4 Human evidence for persistent myeloid activation after myocardial infarction

### 4.1 Hematopoietic and splenic activation in patients

The animal literature supports discrete segments of the interorgan network and identifies the remaining causal gaps. Human evidence for coordinated hematopoietic and splenic activation is suggestive but incomplete. Post-mortem observations support phase-related movement of monocytes from marrow and spleen to infarcted myocardium (14). A PET cohort further showed that marrow inflammatory activity varies with disease context: patients with recurrent MI had worse scar and functional outcomes despite a lower marrow signal than patients with a first MI (63). A single marrow or splenic imaging value should therefore not be interpreted as a clinical signature of trained immunity; it may reflect production, retention, release, or prior depletion and becomes informative only when related to serial blood and cardiac measurements.

Clonal hematopoiesis illustrates the corresponding baseline problem. In chronic ischemic heart failure, increasing DNMT3A- or TET2-mutant clone size was associated with progressively worse long-term mortality (101). Clonal hematopoiesis is not trained immunity and may precede MI by years, but it can alter the hematopoietic substrate on which infarction-induced adaptation occurs. The recent identification of HSC-iM in human samples further shows that inflammatory history can persist in a subset of HSCs and be transmitted to myeloid progeny; these cells were enriched with age and preferentially affected by mutations associated with clonal hematopoiesis (50). Human MI studies must therefore distinguish newly acquired post-MI programming from age-related HSC change, pre-existing inflammatory memory, and clonal selection.

### 4.2 Persistent changes in circulating myeloid cells

Circulating-cell observations provide more direct access to persistent function but remain incomplete. Treated MI survivors have shown prolonged changes in monocyte chemokine-receptor profiles, and patients with premature MI have shown enhanced cytokine responses after ex vivo Toll-like-receptor stimulation together with increased H3K4me3 at the IL10 promoter (4, 5). These findings are compatible with altered innate responsiveness, yet they cannot determine whether the phenotype was induced by MI, existed beforehand, or was maintained by ongoing stimulation. A circulating phenotype becomes more persuasive when persistence beyond the acute phase, altered function after standardized rest-rechallenge, and a plausible metabolic or chromatin substrate converge within a defined cell population.

Transcriptomic machine-learning studies have proposed trained-immunity-related heart-failure biomarkers such as MTURN, but model-derived labels can become circular when the training signature is defined from genes that already reflect inflammation or heart failure (102). Such biomarkers require validation against functional rechallenge, cell composition, and durable metabolic or chromatin changes before they can be interpreted as evidence of innate immune memory.

### 4.3 Cardiac inflammation, fibrosis, and imaging outcomes

Human cardiac observations reach the structural endpoint of the proposed pathway. Longitudinal FAPI-PET/CMR demonstrates fibroblast activation beyond conventional infarct boundaries and relates its spatial extent to remodeling phenotypes (28). Delayed imaging approximately one month after MI likewise detected activated fibroblast volumes that often exceeded the CMR-defined infarct (100), while remote-myocardial T2 signals have been associated with subsequent ventricular remodeling (99). These findings show that biologically active inflammation and fibrosis extend beyond the original necrotic core, but they do not trace those responses back to a durable marrow program. Available observations and the measurements needed to connect them are summarized in **Table 5**.

**Table 5:** Human evidence and recommended measurements for post-MI trained-immunity studies.

Human setting	Sample/timing	Available or required readout	Cardiac linkage	Refs
Treated MI survivors	Serial peripheral blood	Monocyte receptors and inflammatory phenotype	Relate persistence to ventricular volumes/function	(4)
Premature MI without conventional risk factors	PBMC/monocytes	Standardized stimulation, cytokines, H3K4me3	Separate pre-existing susceptibility from MI-associated change	(5)
Human post-mortem MI	Marrow, spleen and myocardium	Regional monocyte abundance and phenotype	Defines interorgan trafficking	(14)
FAPI-PET/CMR cohort	Whole-heart longitudinal imaging	Fibroblast activation and extracellular volume	Links immune phenotype with spatial fibrosis	(28)
Heart-failure biomarker studies	Blood transcriptomics	Candidate trained-immunity signatures	Requires functional and imaging validation	(102)
Acute MI FDG-PET cohort	Early whole-body metabolic imaging	Splenic and bone-marrow FDG uptake	Supports systemic hematopoietic activation but not memory specificity	(62)
Older MI survivors and clonal hematopoiesis	Serial blood with age-stratified sampling	HSC-iM features, clone size, cell composition, rest-rechallenge function	Separate MI-induced change from aging, pre-existing memory, and clonal selection	(50, 101)

### 4.4 What current human studies can—and cannot—establish

Human studies face an unavoidable baseline problem: a phenotype detected after MI may have preceded the event. Dyslipidemia, diabetes, smoking, obesity, chronic infection, psychosocial stress, and clonal hematopoiesis can all alter myelopoiesis and innate responsiveness, while contemporary cardiovascular therapies may modify both immune inputs and remodeling. Cross-sectional observations are therefore informative but cannot determine whether MI induced the phenotype (5).

The central translational requirement is longitudinal evidence. Repeated sampling should determine whether altered innate responsiveness persists after acute necrosis and systemic inflammation have subsided. Functional rest-rechallenge assays in purified monocyte subsets should be interpreted alongside progenitor phenotyping and metabolic or chromatin measurements, rather than a single cytokine, transcript, or histone mark (4).

Cardiac relevance requires the same patients to undergo serial assessment of infarct severity, scar evolution, remote-zone fibrosis, ventricular volumes, and function. A convincing human signal would connect three domains in temporal order: hematopoietic output, persistent innate-cell function, and subsequent cardiac remodeling. This multimodal approach is more informative than labeling any peripheral biomarker as marrow memory in isolation (14, 28).

## 5 Therapeutic implications

Therapeutic implications should be separated along three dimensions: disease phase, biological target, and evidence status. An intervention during acute injury may alter memory establishment but can also impair debris clearance and scar formation; a later intervention may act on marrow maintenance, splenic deployment, or cardiac effector pathways. Candidate trained-immunity-directed approaches should be distinguished from broad anti-inflammatory treatments and from downstream macrophage-fibroblast or ECM strategies. **Table 4** applies this classification and specifies whether support is experimental MI evidence, adjacent mechanistic evidence, indirect human evidence, or clinical experience without demonstrated memory-specific target engagement.

### 5.1 Preventing the establishment of hematopoietic memory

Potential interventions include DAMP/PRR, IL-1/NLRP3, sympathetic, metabolic, and epigenetic pathways. Experimental inhibition of STING, CARD9, ADAM8, and other macrophage signaling nodes improves selected remodeling endpoints in specific models (39, 40, 103, 104). These interventions primarily modify acute or effector-phase inflammation; they do not prove erasure of trained immunity. Early inhibition may reduce excessive recruitment but also compromise clearance and scar integrity. The mixed biological roles of IL-1, IL-6, and macrophage states make broad, prolonged suppression especially risky (1, 35, 41). Patient selection and timing are therefore part of the mechanism, not merely trial logistics.

### 5.2 Modulating persistent bone-marrow and splenic myeloid activity

Intervening at the maintenance stage aims to reverse or contain a durable marrow program after acute repair has begun. Candidate approaches include modulation of metabolic dependencies, epigenetic writers or readers, sympathetic marrow signaling, and myeloid-biased hematopoiesis. This strategy is conceptually attractive because it targets persistence rather than the entire acute inflammatory response, but it carries broad risks: altered host defense, cytopenia, impaired vaccine responses, and disruption of normal hematopoietic adaptation. A maintenance-directed therapy should therefore demonstrate reversal of a defined rest-rechallenge phenotype and altered progenitor output, not merely lower circulating inflammatory markers.

### 5.3 Targeting cardiac myeloid and fibrotic effector pathways

Later strategies could redirect rather than eliminate myeloid responses. During the reparative transition, enhancing efferocytosis, IL-10-associated programs, regulatory lymphocyte signals, or appropriately timed angiogenic and matrix-organizing responses may support resolution while preserving scar stability (60, 68–78). Once a mature scar has formed, local inhibition of persistent macrophage-fibroblast signaling, maladaptive matrix turnover, or remote-zone fibrosis may be safer than suppressing early systemic recruitment. Treg delivery, regulatory B cells, dendritic-cell conditioning, and local RNA or nanoparticle delivery illustrate experimental ways to alter immune-stromal communication (60, 68, 78, 105–107). These are downstream effector strategies, not evidence that hematopoietic memory has been erased.

Timing must therefore be built into the therapeutic hypothesis. In the first several days after MI, studies should prioritize infarct clearance, rupture, scar thickness, and early mortality because apparent anti-inflammatory benefit can conceal impaired healing. In the subacute period, target engagement should include resolution kinetics, monocyte function, fibroblast state, and matrix organization. In the chronic phase, ventricular volumes, remote fibrosis, scar mechanics, recurrent ischemic events, and heart-failure progression become more relevant. A treatment aimed at trained immunity should additionally demonstrate prevention or reversal of a persistent rest-rechallenge phenotype or progenitor bias; a lower cytokine level alone shows anti-inflammatory activity, not memory-specific action.

### 5.4 Balancing infarct repair against long-term remodeling

#### 5.4.1 Therapeutic timing across injury, repair, and chronic remodeling

The same immune pathway can have opposite consequences across the post-MI timeline. During the first days, DAMP sensing and inflammatory recruitment support clearance of necrotic tissue and initiate scar formation. Broad suppression at this stage may reduce inflammatory injury but delay clearance, weaken the scar, or impair host defense. During the reparative transition, the objective shifts toward resolution, angiogenesis, matrix organization, and controlled fibroblast activity. In the chronic phase, persistent myeloid hyperresponsiveness, remote fibrosis, and neurohumoral feedback may become more suitable targets. A drug cannot therefore be evaluated independently of its start time, duration, tissue distribution, and effect on scar mechanics (1, 2, 68).

Interventions can also be divided by whether they block establishment, maintenance, or expression of memory. Reducing metabolic risk or sympathetic input before MI may lower pre-training but is not a practical acute intervention. Blocking an epigenetic writer shortly after MI might prevent durable programming yet carry hematopoietic and immune risks. Later inhibition of a trained effector pathway may be safer but leave the central reservoir intact. Local delivery to cardiac myeloid cells or fibroblasts could limit systemic immunosuppression, whereas marrow-directed therapy may have broader and longer-lasting consequences. These distinctions should guide preclinical endpoints and eligibility criteria rather than being deferred to clinical-trial logistics.

#### 5.4.2 Patient selection and response enrichment

Biomarker enrichment may be essential. Treating all MI survivors assumes that trained immunity is universal, although the strength and even direction of secondary responses are likely heterogeneous. A plausible trial strategy would require evidence of persistent hyperresponsiveness after the acute phase and stratify by infarct severity, metabolic risk, infection history, and remodeling trajectory. Safety monitoring should include infection, cytopenia, delayed healing, ventricular rupture, and impaired vaccine responses. The therapeutic objective is not global erasure of innate memory but selective attenuation of maladaptive persistence while preserving protective recall and tissue repair.

#### 5.4.3 What the colchicine trials do—and do not—show

The colchicine experience is best interpreted through the establishment-maintenance-execution framework. Differences among trials may reflect whether treatment overlapped acute clearance, later inflammatory reinforcement, or chronic risk; whether endpoints captured recurrent ischemic events or structural remodeling; and whether participants carried a persistent myeloid hyperresponsive phenotype (108–115). These trials neither validate nor refute trained-immunity-specific therapy because they did not select patients or verify target engagement using memory-related functional criteria. Instead, they show why the effect of broad anti-inflammatory treatment cannot be assumed to transfer across biological stages and why suppression of contemporaneous inflammation does not demonstrate prevention or reversal of a durable innate-memory program.

#### 5.4.4 Matching target engagement to the treated compartment

Future trials should match target engagement to the biological stage being treated. An establishment-phase intervention should prevent a persistent phenotype from emerging after the acute insult. A maintenance-phase intervention should reverse durable myeloid hyperresponsiveness or progenitor bias. An execution-phase intervention should modify cardiac immune-stromal activity or remodeling. Endpoints should likewise reflect timing, from early scar integrity to later scar composition, ventricular volumes, and function.

**Figure 6** positions intervention classes across pre-MI risk modification, acute inflammation, reparative transition, and chronic heart failure. It emphasizes that the same target may have different consequences when engaged during clearance, scar formation, or chronic remodeling. The highest-value near-term strategy may therefore be biomarker-guided, time-limited intervention in patients with demonstrable persistent myeloid hyperresponsiveness rather than unselected long-term anti-inflammatory therapy.

**Figure 6:**
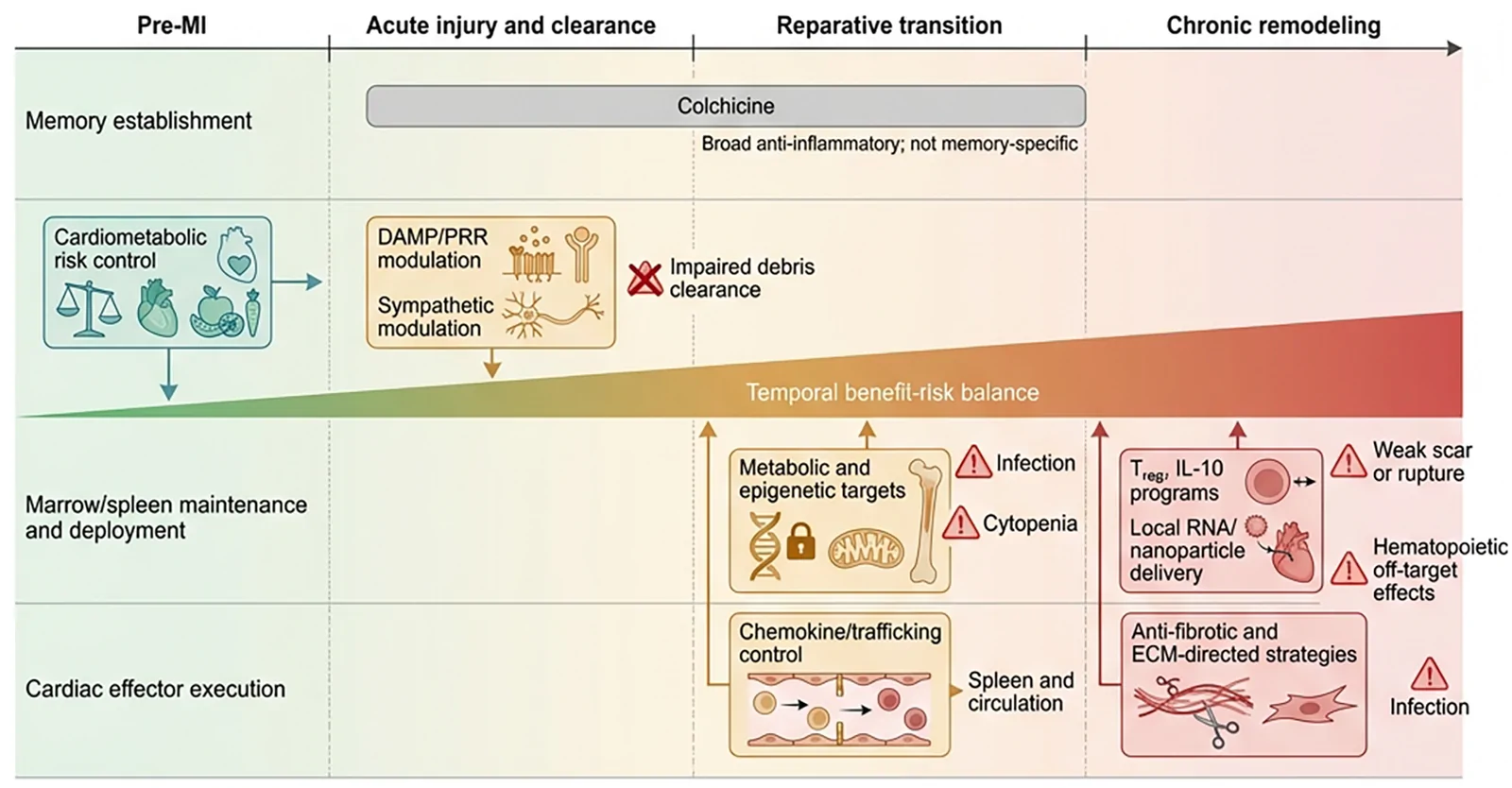
Therapeutic opportunities and risks across the post-MI trained-immunity life cycle: candidate interventions are mapped to memory establishment, marrow or splenic maintenance and deployment, and cardiac effector activity. Because early innate responses support clearance and scar formation, therapeutic effects depend on timing and compartment. Colchicine is shown as a broad anti-inflammatory example rather than a memory-specific treatment. MI, myocardial infarction.

## 6 Unresolved questions and future directions

### 6.1 Distinguishing trained immunity from persistent stimulation and pre-existing myeloid bias

The first challenge is to determine what produces persistence. A phenotype detected after MI may reflect continuing necrosis, recurrent ischemia, neurohumoral activation, altered cell composition, a pre-existing cardiometabolic bias, or durable reprogramming induced by the infarct. Repeated sampling after acute signals have declined, combined with defined rest-rechallenge assays and lineage-resolved molecular measurements, is needed to separate these possibilities. Infarct size, reperfusion, metabolic disease, infection, clonal hematopoiesis, and heart-failure progression must be treated as biological modifiers rather than background covariates.

### 6.2 Establishing causal continuity across the heart-hematopoietic-heart network

Future experimental studies will benefit from examining the full pathway within the same model. Lineage tracing, marrow transplantation, barcoding, parabiosis, and cell-specific perturbation can be combined with single-cell RNA and chromatin profiling to follow hematopoietic progeny from marrow and spleen into the heart. Spatial transcriptomics and multiplex imaging can then place these cells in relation to fibroblast states, vascular structures, cardiomyocyte stress, and regional matrix organization. Serial imaging and quantitative assessment of scar composition, cross-linking, ventricular volumes, and function will connect cellular findings to organ-level remodeling.

This integrated approach is particularly important because the consequences of inflammation depend on timing. Early myeloid recruitment supports clearance and scar stabilization, whereas delayed resolution or recurrent activation may promote protease activity, disordered matrix turnover, remote fibrosis, and chamber dilatation. Experimental interventions should therefore report initial infarct size, rupture and healing outcomes, later scar mechanics, and ventricular function so that protection from chronic remodeling can be distinguished from changes in the initial injury.

Three complementary designs can test these hypotheses without assuming that a repeat-infarct model is already established. First, MI can serve as the training exposure, followed after recovery by a standardized heterologous challenge ex vivo or *in vivo*. Purified HSPCs or monocytes should be rested before stimulation with a defined Toll-like-receptor ligand, beta-glucan, oxidized LDL, or adrenergic signal. Persistent, stimulus-calibrated hyperresponsiveness after acute MI signals have declined would test Gate 1 without the anatomical and survival confounding introduced by a second infarct.

Second, marrow or purified HSPCs from MI-exposed and sham-treated donors can be transplanted into naive recipients after the donor acute phase has resolved. Recipients can then undergo the same standardized MI or ischemia-reperfusion protocol. Donor-derived lineage tracking, cardiac macrophage and fibroblast profiling, scar composition, ventricular volumes, and function would connect progenitor memory to a later cardiac outcome. Matching recipient injury severity and interrupting a candidate memory mechanism before transplantation would help distinguish transferred memory from differences in the initial infarct. This design most directly links all three gates while avoiding the ambiguity of two infarcts in the same animal.

Third, a two-hit ischemia-reperfusion model could be used as an exploratory stress test, with a small, controlled index injury followed after stable recovery by a second standardized insult. The design is clinically intuitive but difficult to interpret because the first infarct changes ventricular geometry, coronary anatomy, scar mechanics, neurohumoral tone, and survival. Sham first-hit controls, matched area at risk, reperfusion, infarct size, and hemodynamics are essential. Parallel selective disruption of splenic macrophage-VCAM1 retention or splenic hematopoiesis, without removing direct marrow recruitment, would determine whether the spleen is required, amplifying, or dispensable in each design (56, 57).

### 6.3 Priorities for experimental and human validation

The highest-priority studies are those that connect more than one interorgan handoff in the same model or patient. Prospective MI cohorts should combine repeated assessment of hematopoietic output and purified-monocyte rest-rechallenge responses with selected metabolic or chromatin measurements and serial cardiac imaging (4, 5, 14, 28). Such studies should test whether a persistent cellular phenotype temporally precedes scar expansion, remote-zone fibrosis, ventricular dilatation, or functional decline, while accounting for infarct severity, reperfusion, medication, metabolic disease, infection, and clonal hematopoiesis.

### 6.4 Aging as a modifier of hematopoietic memory and repair

Among the biological modifiers that may complicate these experimental and clinical validation strategies, aging is particularly important because it affects both the baseline hematopoietic state and the cardiac response to injury. Older HSCs show reduced regenerative reserve, myeloid-biased output, altered metabolic and epigenetic regulation, and greater exposure to chronic inflammatory signals. The human HSC-iM study found that inflammatory-memory HSCs accumulate with age and are preferentially affected by mutations associated with clonal hematopoiesis (50). An older patient may therefore enter MI with pre-existing inflammatory memory, clonal selection, or both, making a post-MI phenotype less attributable to the infarct alone.

Aging also changes downstream execution. Immunosenescence can impair pathogen defense and vaccine responses, whereas inflammaging can raise basal myeloid activation. Reduced efferocytosis, delayed resolution, endothelial dysfunction, fibroblast senescence, altered collagen turnover, and increased matrix stiffness may change how the same hematopoietic output affects scar formation and remote myocardium. These processes can increase adverse remodeling while narrowing the safety margin for anti-inflammatory or marrow-directed treatment. Age should therefore be treated as a biological variable rather than a demographic adjustment: preclinical studies should include young and aged animals, and clinical cohorts should assess clonal hematopoiesis, comorbidity, medication, baseline inflammatory state, cell composition, and functional reserve when defining trained-immunity phenotypes. These age-related differences also have direct implications for the timing, efficacy, and safety of interventions targeting hematopoietic or inflammatory pathways after MI.

### 6.5 Toward temporally informed intervention

Translation will depend on matching treatment to the biological phase of disease. Interventions during memory establishment may reduce the imprint of acute injury but must preserve debris clearance and scar formation. Maintenance-directed approaches may target metabolic, epigenetic, or neurohematopoietic pathways after the early reparative phase. Effector-directed strategies may act locally on macrophage-fibroblast communication, matrix turnover, or chronic inflammatory recruitment. Biomarker-guided selection and time-limited treatment may therefore offer a more favorable balance than uniform long-term immunosuppression. **Figure 7** summarizes a future research pathway linking longitudinal immune phenotyping, hematopoietic lineage analysis, spatial cardiac profiling, and remodeling outcomes. Progress across these areas will clarify how frequently trained immunity contributes to post-MI remodeling and whether it can be modified without compromising protective innate responses.

**Figure 7:**
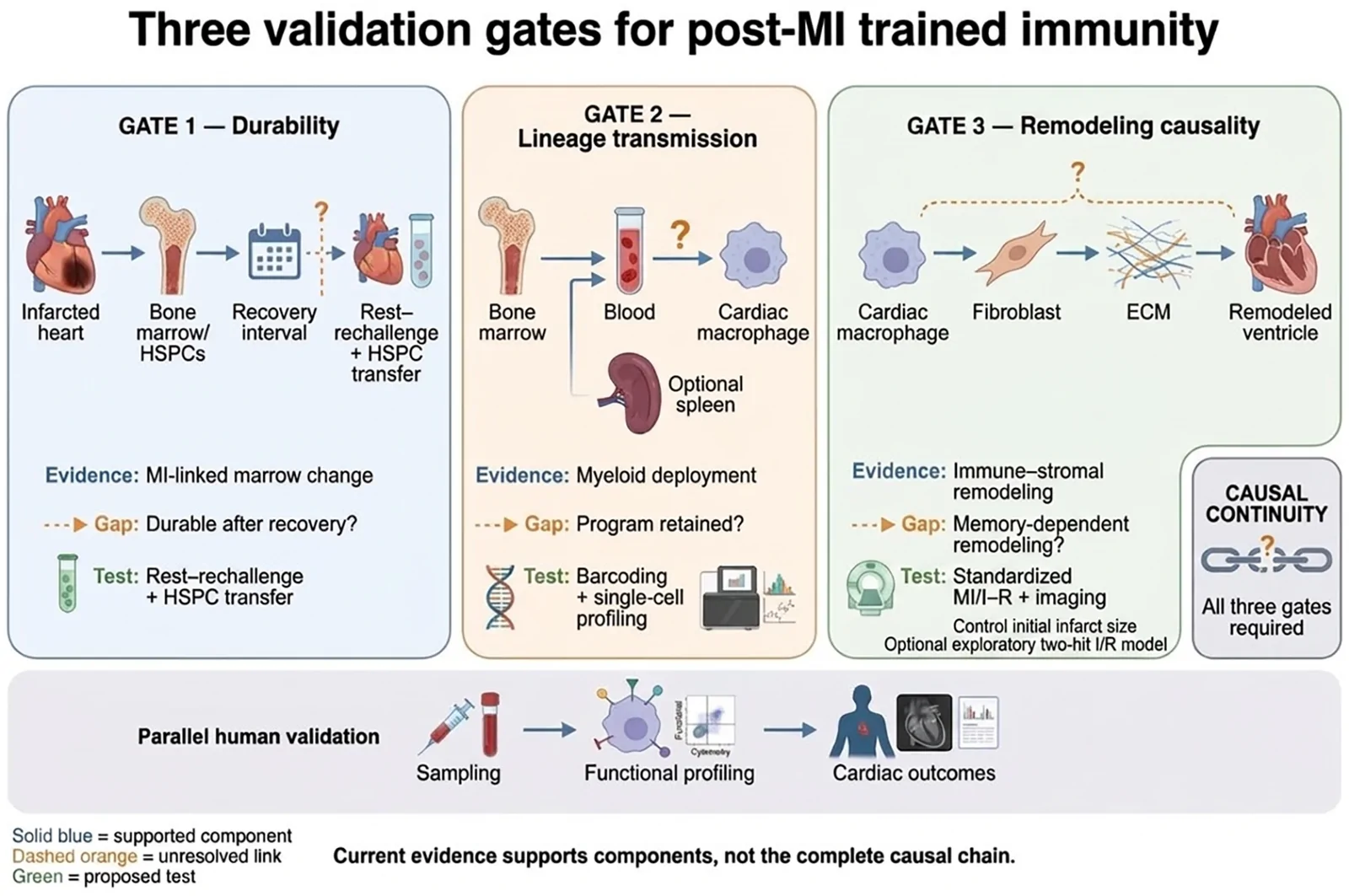
Three validation gates and a parallel human-validation track for testing causal continuity in the proposed post-MI trained-immunity network: gate 1 asks whether an MI-induced HSPC program persists after acute signals resolve. Gate 2 asks whether altered function is retained during direct marrow-to-blood-to-heart recruitment or optional splenic amplification that rejoins the circulation. Gate 3 asks whether lineage history modifies cardiac macrophage–fibroblast–ECM interactions and ventricular remodeling independently of initial infarct size. Each gate pairs existing evidence and an unresolved link with a proposed test, including rest–rechallenge, HSPC transfer, lineage barcoding, single-cell profiling, standardized MI or ischemia–reperfusion, and an exploratory two-hit model. The human-validation track links longitudinal immune profiling with cardiac outcomes. Solid blue denotes supported components, dashed orange unresolved links, and green proposed tests. The gates are validation requirements, not established causal steps.

## 7 Conclusions

The persistence of adverse remodeling after a transient ischemic insult poses a temporal problem that cannot always be explained by the lifespan of circulating myeloid cells or by a self-limited local inflammatory response. Trained immunity offers a plausible solution: myocardial injury may reprogram bone-marrow hematopoiesis; altered myeloid progeny may reach the heart directly or be retained, expanded, and deployed through the spleen; and cardiac macrophage-fibroblast-ECM interactions may convert this systemic bias into lasting structural change. Evidence supports these components to different degrees, but no study has yet demonstrated complete causal continuity from MI-induced progenitor memory to adverse ventricular remodeling. Aging, pre-existing inflammatory memory, and clonal hematopoiesis further modify this network. Establishing when durable memory is present, whether the spleen is required or amplifying, and which phase can be targeted safely will determine whether trained immunity is a driver, a context-dependent amplifier, or an accompanying feature of post-MI remodeling.
